# The Role of Decidual Subpopulations in Implantation, Menstruation and Miscarriage

**DOI:** 10.3389/frph.2021.804921

**Published:** 2021-12-23

**Authors:** Joanne Muter, Chow-Seng Kong, Jan J. Brosens

**Affiliations:** ^1^Division of Biomedicine, Warwick Medical School, University of Warwick, Coventry, United Kingdom; ^2^Tommy's National Centre for Miscarriage Research, University Hospitals Coventry and Warwickshire NHS Trust, Coventry, United Kingdom

**Keywords:** endometrium, implantation, miscarriage, decidualization, senescence, innate immunity, menstruation

## Abstract

In each menstrual cycle, the endometrium becomes receptive to embryo implantation while preparing for tissue breakdown and repair. Both pregnancy and menstruation are dependent on spontaneous decidualization of endometrial stromal cells, a progesterone-dependent process that follows rapid, oestrogen-dependent proliferation. During the implantation window, stromal cells mount an acute stress response, which leads to the emergence of functionally distinct decidual subsets, reflecting the level of replication stress incurred during the preceding proliferative phase. Progesterone-dependent, anti-inflammatory decidual cells (DeC) form a robust matrix that accommodates the conceptus whereas pro-inflammatory, progesterone-resistant stressed and senescent decidual cells (senDeC) control tissue remodelling and breakdown. To execute these functions, each decidual subset engages innate immune cells: DeC partner with uterine natural killer (uNK) cells to eliminate senDeC, while senDeC co-opt neutrophils and macrophages to assist with tissue breakdown and repair. Thus, successful transformation of cycling endometrium into the decidua of pregnancy not only requires continuous progesterone signalling but dominance of DeC over senDeC, aided by recruitment and differentiation of circulating NK cells and bone marrow-derived decidual progenitors. We discuss how the frequency of cycles resulting in imbalanced decidual subpopulations may determine the recurrence risk of miscarriage and highlight emerging therapeutic strategies.

## Introduction

The human endometrium is defined by its ability to execute opposing functions, often simultaneously, and to transition seamlessly between different physiological states. For example, menstrual shedding occurs in parallel with activation of repair mechanisms (1, 2), optimal fertility depends on the receptive endometrium engaging in embryo selection and rejection (3), and pregnancy requires transformation of a cycling mucosa into a robust, semi-permanent matrix capable of accommodating the placenta throughout gestation (4). The remarkable capacity of the human endometrium to switch effortlessly between states and functions on a cyclical basis is shared only with a handful of other menstruating mammals, including higher primates, some species of bats, and the elephant shrew (5, 6). Menstruating mammals share several other reproductive characteristics, such as spontaneous ovulation, a hemochorial placenta characterised by deep invasion of the maternal arteries by placental trophoblast, and birth of only 1 or 2 well-developed offspring per pregnancy (6). Further, three distinct features set the non-pregnant endometrium of menstruating species apart from other mammals: rapid proliferation and tissue growth, accumulation of uterine natural killer (uNK) cells, and spontaneous decidualization of stromal cells (5).

Decidualization of endometrial stromal cells is a multistep differentiation programme that starts with an evolutionarily conserved acute cellular stress response (7), which results after several days in the emergence of specialist decidual cells (4, 8). In pregnancy, decidual cells cooperate with local immune cells to form a specialist matrix for controlled trophoblast invasion and placenta formation (9, 10). Decidualization occurs in all mammalian species where implantation involves breaching of the luminal endometrial epithelium by the conceptus, but the extent of the decidual reaction varies markedly and correlates to the depth of trophoblast invasion in each species (11). Importantly, decidualization and accumulation of uNK cells depend on signals emanating from the implanting embryo in most mammals (12). However, in menstruating species both processes are initiated in each cycle, irrespective of an implanting embryo (5, 6). As we will discuss later, this switch from embryonic to maternal control over the decidual process not only accounts for the evolution of menstruation but also bequeaths the endometrium with a robust mechanism to reject low-fitness embryos. In human endometrium, the initial decidual stress response coincides with the opening of the midluteal implantation window, whereas the emergence of morphologically differentiated decidual cells (DeC), characterised by abundant cytoplasm and enlarged nuclei, heralds the closure of the 4-day implantation window (4).

The endometrium is often viewed as an effector tissue solely under the control of the rise and fall in ovarian oestrogen and progesterone production and, consequently, capable of carbon-copying itself from cycle-to-cycle. The discovery that endometrial regeneration and tissue homeostasis are critically dependent on bone marrow-derived, non-hematopoietic progenitor cells and innate immune cells has all but torpedoed this historical misconception (13–17). Further, novel technologies, such as single-cell RNA-sequencing and endometrial organoid models, are revealing how oestrogen (E2)-dependent proliferation during the follicular phase controls the specification of endometrial epithelial and stromal cells into different subpopulations with distinct functions following ovulation (8, 18, 19). Here, we summarise recent insights into how distinct decidual subpopulations bestows the endometrium with the ability to become receptive, but also selective at implantation, to trigger menstrual breakdown while activating repair mechanisms, and to transition successfully into a gestational tissue. Furthermore, we highlight the importance of dyshomeostasis of decidual subpopulations in recurrent miscarriage and touch upon the ensuing therapeutic opportunities.

## Ontogenesis of Spontaneous Decidualization

Mammalian genes are highly conserved. Consequently, evolution relies largely on changes in non-coding, regulatory sequences that alter gene expression (20). A major mechanism driving reproductive diversity in mammals involves incorporation of transposable elements (TEs) in regulatory DNA sequences, which in turn leads to rewiring of signal transduction pathways and transcription factors (TFs) to drive expression of novel gene networks. TEs comprise a vast array of DNA sequences that can, or could, move to new sites in genomes, either by a “cut-and-paste” mechanism (transposons) or through “copy-and-paste” RNA intermediates (retrotransposons). In contrast to genes, TEs are highly variable and frequently species-specific (20). Colonisation of mammalian genomes with *MER20*, a 'cut-and-paste' DNA transposon, coincided with the emergence of decidualization and invasive placentas. *MER20* elements encode 13% of the putative enhancers of genes that gained expression in DeC of eutherian (placental) mammals, including higher primates and humans (21, 22). Further, emergence of menstruation in the primate lineage occurred in parallel with genomic integration of *Alu* retrotransposons harbouring a triple TF binding motif (oestrogen receptor-, basic leucine zipper-, and PAX domain-binding sequences) (23), indicating that TEs also governed the evolution of spontaneous decidualization. Based on comparative transcriptomics, hundreds of genes have now been identified in pregnant endometrium that were gained or lost in primate and human lineages (24). Emerging evidence suggests that decidual genes recruited recently in the human lineage play a disproportionate role in prevalent pregnancy disorders, including early pregnancy loss and preterm birth (24).

**Figure 1:**
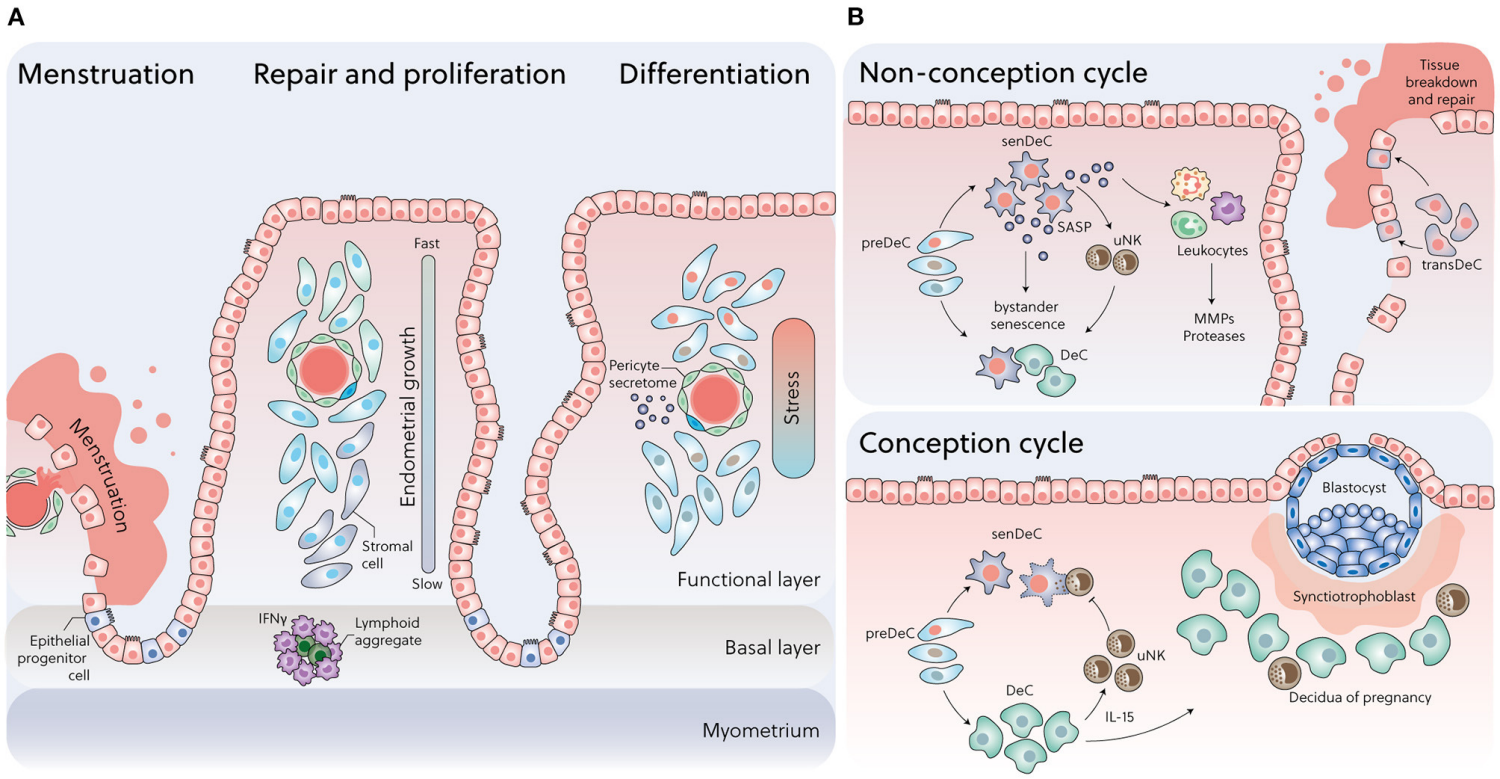
Dynamic changes in cell states across the menstrual cycle. **(A)** Following menstruation, rapid re-epithelization of the endometrium may involve mesenchymal-epithelial transition (MET), proliferation of epithelial progenitors from unshed areas, or both. Positional oestrogen-dependent proliferation, reflecting an IFN-γ gradient generated by lymphoid aggregates residing in the endometrial-myometrial junction, imposes various levels of replication stress on individual cells during the follicular phase, which in turn drives the divergence in cell states upon decidualization. **(B)** Upper panel: In a non-conception cycle, falling progesterone levels promote senDeC/transDeC dominance and bystander senescence via prolonged SASP secretion, resulting in leukocyte recruitment and menstrual breakdown. Lower panel: Upon successful embryo implantation, progesterone-dependent DeC cells control rapid clearance of senDeC by recruiting and activating uNK cells.

Despite these genomic adaptations, human endometrial stromal cells are not intrinsically capable of differentiating into DeC. Instead, spontaneous decidualization in response to hormonal signalling is an endometrial trait that emerges at some point after the menarche. For example, the endometrium in most term foetuses and neonates is only weakly proliferative, despite prolonged exposure to very high concentrations of unbound estrogens and progesterone *in utero*. While secretory changes in endometrial glands can be observed occasionally at birth, decidual or menstrual changes are rare (25). These findings from post-mortem studies are corroborated by the observation that overt neonatal uterine bleeding, defined as menstruation-like bleeding triggered by a rapid fall in circulating sex hormones of maternal origins, affects only 4–5% of female babies during the first week of life (26). Progesterone responsiveness of the endometrium becomes established after prolonged E2-dependent growth of the uterus, which starts before breast development in pre-pubertal girls and continuous after the menarche (27). The dependency of spontaneous decidualization on E2-dependent hyperproliferation is also apparent in the spatial organisation of this process in cycling endometrium. Following menstruation, proliferation of glandular epithelial and stromal cells accelerates with increasing distance from the endometrial-myometrial interface and peaks on cycle day 10 in the upper one third of the superficial endometrial layer (Figure 1A) (28). This positional proliferative response has been linked to presence of lymphoid aggregates residing in the basal endometrial layer (29–31), which purportedly secrete interferon gamma (IFN-γ), a potent inhibitor of steroid hormone responses (32). Thus, as the endometrium grows beyond the local IFN-γ gradient, cellular proliferation accelerates quickly, thereby imposing various levels of replication stress on individual cells (Figure 1A). After the postovulatory rise in progesterone levels, proliferation of glandular epithelial cells first decreases and then ceases altogether in concert with the onset of apocrine glandular secretions, heralding the start of the midluteal window of implantation (33). Concurrently, proliferating uNK cells accumulate while stromal cells in the proximity of the luminal epithelial exit the cell cycle and start decidualizing (4). Pericytes surrounding the terminal spiral arterioles in the superficial layer also undergo morphological changes that are characteristic of a decidual response. Pericytes are not only biophysically and metabolically different from their stromal counterparts (34, 35), but produce a distinct decidual secretome, rich in chemokines and cytokines implicated in trophoblast migration and intravascular invasion (36) (Figure 1A).

Thus, across the menstrual cycle, the spatiotemporal responses of the endometrium to ovarian hormones are tightly controlled by changes in cell cycle status. How hyperproliferation of stromal cells is linked to spontaneous decidualization and subsequent endometrial fate decisions has been elusive until recently. As we will describe next, recent single-cell transcriptomic studies and modelling of the menstrual cycle in 3D cultures uncovered compelling evidence that human endometrium exploits rapid proliferation and replicative exhaustion to generate functionally distinct subpopulations that critically determine endometrial fate decisions following the implantation window (8, 18, 37).

## Specification of Decidualizing Cells Into Functionally Distinct Subpopulations

### Decidual States

Decidualization is not a binary differentiation response. Instead, it can be viewed as a triphasic process that starts with an acute inflammatory stress response, which is followed first by an anti-inflammatory phase and then a second, irreversible inflammatory state. As mentioned, the initial inflammatory decidual response maps to the midluteal implantation window (4). In non-conception cycles, the subsequent anti-inflammatory decidual phase is brief as falling progesterone levels promote rapid transition to the irreversible inflammatory state, which precedes menstrual breakdown (38). Upon successful embryo implantation, however, the anti-inflammatory decidual phase is massively prolonged and maintained for much of the pregnancy, although the decidua ultimately assumes a pro-inflammatory state prior to parturition (39, 40). The observation that DeC switch phenotype prior to the onset of labour in the absence of a discernible fall in circulating progesterone levels led to hypothesis that the timing of birth is determined by a “decidual clock” (41, 42). Thus, the term “decidualization,” which is derived from the Latin verb “decidere” (to die, to fall off or to detach), aptly describes the physiological process that links the window of implantation to tissue destruction associated with menstruation and parturition.

It should be obvious that parsing the mechanisms that enable DeC to switch phenotypes is not merely of academic interest but fundamental for our understanding of the physiological processes that control embryo implantation, menstruation and parturition. Valuable insights into this process emerged from a simple reconstruction of the decidual pathway in cultured primary endometrial stromal cells using single-cell transcriptomics (8). This analysis revealed that differentiating stromal cells undergo extensive and coordinated transcriptional reprogramming during the initial inflammatory decidual phase, triggered by rising intracellular cyclic adenosine monophosphate (cAMP) and activation of decidua-specific TFs that interact with the liganded progesterone receptor (43–45). Transcriptional reprogramming of stromal cells involves an acute cellular stress response, which starts with a burst reactive oxygen production and secretion of inflammatory mediators and nuclear alarmins, including interleukin 33 (IL-33) and high mobility group box 1 (HMGB1) (13, 46, 47). In parallel, wholesale remodelling of the chromatin landscape, involving opening, as well as closure of numerous DNA loci, enables decidual TF complexes to gain access to promoter and enhancer regions that control the expression of specific decidual gene networks (23, 48, 49). Cells transitioning through this preparatory phase are denoted pre-decidual cells (preDeC). Most reprogrammed cells emerge as progesterone dependent, anti-inflammatory DeC. However, inflammatory reprogramming also compounds DNA damage already present stromal cells burdened by replication stress, which in turn gives rise to a discrete population of senescent decidual cells (senDeC; Figure 1B) (8, 13).

### Anti-inflammatory Decidual Cells

Progesterone-dependent DeC are typified by activation of cellular defence mechanisms and selective silencing of stress-responsive signalling pathways (summarised in Table 1). Consequently, DeC are not only protected against oxidative and metabolic stress but also largely impervious to noxious environmental cues. Silencing of stress pathways is also critical for continuous progesterone signalling in DeC (51). Through tight intercellular connexions (58), DeC form a robust matrix, which in pregnancy accommodates invading extravillous trophoblast and local immune cell populations (9). Although the decidual secretome is largely devoid of inflammatory mediators, they produce an abundance of C-X-C motif chemokine ligand 14 (CXCL14) and IL-15, essential for uNK cell chemotaxis and activation, respectively (8, 59). By contrast, epigenetic silencing of other chemokines precludes infiltration of the decidual matrix by cytotoxic T lymphocytes (60).

**Table 1:** Mechanisms of DeC cellular defence and stress-resistance.

**Pathway**	**Description**	**References**
Cessation of circadian rhythms	Decidual loss of Period 2 (PER2) expression silences circadian gene expression in differentiating EnSCs, matching aperiodic gene expression in the implanting conceptus.	(50)
Oxidative stress resistance	Induction of various free radical scavengers upon decidualization including SOD2, monoamine oxidases A and B, thioredoxin, glutaredoxin and peroxiredoxin confer oxidative stress resistance to decidual cells. Upregulation of mitogen-activated protein phosphatase 1 (DUSP1) silences c-Jun NH-terminal kinase (JNK) stress signalling, and inhibits FOXO3a, a forkhead proteins implicated in oxidative cell death.	(51, 52)
Uncoupling of stress signals and SUMOylation	Decidualization imposes a stress resistant global cellular hypoSUMOylation state by modulation of various SUMO-specific ligases and proteases, preventing PGR transcriptional repression.	(53, 54)
Silencing of phospholipase C signalling	Induction of phospholipase C (PLC)-related catalytically inactive protein 1 (PRIP-1) uncouples PLC activation from intracellular Ca^2+^ release by attenuation of inositol triphosphate (IP3) signalling.	(55)
Resistance to microRNA mediated gene silencing	Downregulation of argonaut proteins (AGO1 and AGO2) upon decidualization renders the endometrium resistant to microRNA mediated gene silencing.	(56)
Downregulation of O-linked N-acetylglucosamine (O-GlcNAc) posttranslational modification	Decidualization of EnSCs results in reduced global O-GlcNAcylation, mediated by decreased expression of the metabolic stress enzyme O-GlcNAc transferase (OGT), without changes in its reciprocal mediator O-GlcNAcase (OGA)	(57)

### Senescent Decidual Cells

In virtually all aspects, senDeC are the functional opposites of DeC. Senescence denotes a cellular stress response triggered by telomere shortening and replicative exhaustion as well as a myriad of other stressors that cause macromolecular damage (61). Activation of tumour suppressor pathways and upregulation of cyclin-dependent kinase inhibitors p16^INK4a^ and p21^CIP1^ lead to permanent cell cycle arrest, resistance to apoptosis, de-repression of retrotransposons, and production of a bioactive secretome, referred to as the senescence-associated secretory phenotype (SASP) (62). The composition of the SASP is tissue-specific and typically includes proinflammatory and immuno-modulatory cytokines, chemokines, growth modulators, angiogenic factors, and extracellular matrix (ECM) proteins and proteases (63). Senescence has been described as an evolutionarily conserved cellular programme with both beneficial and detrimental effects (62). For example, acute senescence, characterised by transient SASP production and rapid immune-mediated clearance of senescent cells, is also widely implicated in processes involving physiological tissue remodelling, including during foetal development, placenta formation and wound healing (61, 64). By contrast, persisting senescent cells cause chronic, sterile inflammation, also known as “inflammaging” (63), a pathological state that underpins ageing and age-related disorders. In the endometrium, senDeC are characterised by their abounding capacity to initiate tissue remodelling, mediated by a SASP rich in ECM proteins and proteinases, angiogenic modulators, growth factors, and chemokines involved in neutrophil migration (18). Compared to undifferentiated stromal cells, DeC appear highly susceptible to bystander senescence, meaning that they acquire a senescent phenotype upon prolonged SASP exposure (13, 65, 66). When extrapolated to the *in vivo* situation, these observations suggest that in the absence of effective immune cell surveillance, cellular senescence is poised to propagate across the susceptible superficial endometrial layer, rendering tissue breakdown in a piecemeal fashion inevitable (1, 67).

### Transitional Decidual Cells

Reconstruction of the decidual pathway in endometrial assembloids, consisting of gland-like organoids and primary stromal cells, not only confirmed the divergence of differentiating stromal cells into anti-inflammatory DeC and pro-inflammatory senDeC but also revealed a third decidual subpopulation with hallmarks of mesenchymal-epithelial transition (MET) (18). Computation predictions based on gene expression indicate that these cells, termed transitional DeC (transDeC), are highly autonomous, i.e., largely devoid of receptors and ligands that mediate interactions with other decidual subpopulations. Enrichment of gene ontology categories, such as “regulation of stem cell proliferation,” “blood vessel development,” and “wound healing,” further suggests that transDeC are poised to effect tissue repair (18), which is in line with experimental evidence that MET drives re-epithelisation of the endometrium following menstruation and parturition (68, 69).

Pharmacological elimination of pre-stressed stromal cells in 3D assembloids blunts the initial decidual inflammatory response, which in turn massively accelerates the emergence of DeC at the expense of senDeC and, to a lesser extent, transDeC (18). This observation is pivotal as it demonstrates the importance of E2-dependent hyperproliferation and replicative exhaustion during the proliferative phase in determining the amplitude of the pre-decidual inflammatory response and the subsequent balance between DeC, and senDeC/transDeC. Thus, the *in vitro* data suggest that following rapid E2-dependent proliferation, the default trajectory of the decidual pathway is inevitably towards tissue destruction and repair. As DeC are sensitive to bystander senescence, they can escape this fate during a narrow window only by engaging innate immune cells to eliminate their senescent counterparts (Figure 1B) (8).

## Endometrial Homeostasis During the Luteal Phase

Although 2D and even 3D cultures are highly reductionist models to study *in vivo* events, several aspects of the *in vitro* decidual pathway are recapitulated *in vivo*. Tissular cAMP levels increase markedly in the endometrium following the postovulatory rise in progesterone levels (70). While the nature of the *in vivo* ligand(s) responsible for adenylyl cyclase activation in stromal cells has been debated for years, recent evidence firmly implicates prostaglandin E2 (PGE2) as the ancestral deciduogenic signal (71). As a consequence of cAMP-dependent protein kinase A activation and progesterone signalling, proliferation of epithelial and stromal cells in the superficial endometrial layer ceases and differentiation is initiated (72). In parallel, senescence-associated b-galactosidase (SAbG) activity in whole endometrial biopsies increases sharply following ovulation and levels continue to rise upon progression from the early- to late-luteal phase (13). SAbG activity, reflecting lysosomal mass (73), is a widely used biomarker of senescent cells, although it lacks specificity (62, 74). However, transition from proliferative to secretory phase also coincides with the emergence of other canonical senescence markers in the endometrium, including loss of lamin B1, induction of the tumour suppressor p53, the cyclin-dependent kinase inhibitor p16^INK4a^, and senescence-associated histone modifications (13, 75). Spatiotemporal profiling of p16^INK4a^-positive cells in 308 timed endometrial biopsies demonstrated that senescent cells are much more abundant in luminal when compared to glandular epithelium during the midluteal window of implantation. In the stroma, ~1% of cells are p16^INK4a^-positive in the early-luteal phase. Their abundance rises transiently during the window of implantation, which is followed by a much steeper increase in premenstrual endometrium (13).

The temporal profile p16^INK4a^-positive cells in the endometrium highlights the important role for continuous progesterone signalling in constraining cellular senescence. Perhaps not surprisingly, this task is executed primarily by uNK cells, the most abundant immune cells in luteal phase endometrium and the decidua of pregnancy (Figure 1B) (13, 19, 37, 76). Both glandular epithelial cells and DeC secrete chemokines, most prominently CXCL14, involved in recruiting circulating NK cells into the endometrium (77, 78). This multifaceted chemokine also plays an important role in immunosurveillance for bacterial and viral infections (79). Once recruited, NK cells are subjected to progressive differentiation, a process under the control of DeC through secretion of IL-15 (80). Maturation of uNK is characterised by sequential acquisition of killer cell immunoglobulin-like receptors (KIRs) and CD39 on the cell surface. Immature uNK (KIR^−^CD39^−^) display higher proliferative capacity in response to IL-15, which diminishes with increasing maturity (80, 81). The mature uNK (KIR^+^CD39^+^) subset is characterised by increased production of cytotoxic granzyme-A (80). Single-cell analyses confirmed the presence of three transcriptionally distinct uNK subsets in both luteal endometrium and the maternal-foetal interface in early pregnancy (8, 10).

In primary cultures, uNK cells target and kill senDeC with exquisite precision and efficacy (13, 37). Clearance of senDec is achieved primarily through granule exocytosis, in which the uNK cells physically engage with target cells and deliver cytolytic granules containing perforin, granzyme A and granzyme B (13, 82). How uNK cell selectively target senDeC is not fully understood, although experimental evidence implicates activation of killer cell lectin like receptor K1 [KLRK1, also known Natural Killer Group 2 member D (NKG2D)] (83). This activating uNK cell receptor binds stress-induced ligands present on the surface of stressed and senescent cells. These ligands belong to the MHC class I chain-related protein (MIC) and unique-long 16 binding protein (ULBP) families of proteins (84, 85). SASP metalloproteinases, such as ADAM metallopeptidase domain 9 (ADAM9), ADAM10, and ADAM17, can cleave stress-induced ligands from the cell surface, thereby enabling senescent cells to evade immune recognition (86, 87). However, preDeC and DeC firmly block senDeC from activating this escape mechanism by secreting an abundance of TIMP metallopeptidase inhibitor 3 (TIMP3), a potent inhibitor of metalloproteinases (88). IL15, CXCL14 and TIMP3 are already highly expressed by preDeC during the midluteal phase, meaning that immune surveillance of damaged and senescent cells is operational during the implantation window.

The uNK cell-DeC partnership is critically dependent on continuous progesterone signalling, which explains, at least in part, why the endometrium switches dramatically to a pro-inflammatory state prior to menstruation. In a conception cycle, however, sustained uNK cell-DeC cooperation occurs alongside recruitment of circulating bone marrow-derived decidual progenitor cells (8, 14). Decidual progenitor cells in luteal phase endometrium are clonogenic cells poised for rapid proliferative expansion in early pregnancy. Unlike resident endometrial stromal cells, decidual progenitors highly express *PRL*, which encodes the canonical *in vitro* decidual marker prolactin (14, 16). Thus, endometrial homeostasis upon interstitial embryo implantation and subsequent transformation into the decidua of pregnancy are critically dependent upon successful recruitment of non-uterine cells; i.e., circulating NK cells and non-hematopoietic bone marrow-derived mesenchymal progenitor cells (8, 13, 14, 16, 37).

## Implantation: Endometrial Receptivity and Selectivity

### The Implantation Paradigm

It is widely assumed that breaching of the endometrial surface (luminal) epithelium by the blastocyst is the critical, rate-limiting step during human implantation. This implantation paradigm, which is based on studies in mice and other animal models (89–91), assumes that the luminal epithelium is a robust barrier that only transiently expresses the machinery needed for embryo apposition, attachment and invasion. In other words, transient changes in luminal epithelium are believed to define the boundaries of the implantation window. A potential problem with this paradigm is that it glosses over distinct inter-species differences and reproductive challenges. Mice are litter-bearing mammals and the barrier function of the luminal epithelial is critical for synchronised implantation of multiple blastocysts. Murine embryos in the uterine cavity can temporarily arrest in development (diapause) while awaiting a transient surge in circulating oestrogen levels, which simultaneously renders the endometrium receptive and activates dormant embryos for implantation (92). Oestrogen levels also rise transiently during the midluteal phase of the menstrual cycle but there is no evidence that it serves as a nidation signal (93, 94). In contrast to mice, human conception involves a single blastocyst, which often harbours complex chromosomal errors and lacks the ability to enter diapause (95, 96). Further, the luminal epithelium during the midluteal phase consists of a patchwork of p16^INK4a^-positive and -negative epithelial cells (13). While yet untested, p16^INK4a^-positive senescent epithelial cells plausibly create areas of little or no resistance to embryo implantation. It is indeed notable that apposition and attachment of blastocysts to luminal epithelium have been observed in many species, but histological evidence of this implantation stage has not yet been documented in humans (97). Our assertion that the barrier function of the human endometrium is degraded does not imply that luminal epithelial cells are dispensable for implantation. For example, the luminal epithelium mediates progesterone-dependent absorption of uterine fluid (98, 99), which critically ensures “closure” of the uterine cavity during the implantation window (100). In co-cultures, contact between human blastocysts and endometrial epithelial cells induces an embryonic transcriptional response that may promote further implantation (101).

Amongst primates, only humans and apes exhibit primary interstitial implantation, where the entire conceptus is drawn into the endometrium (97). Rather than reflecting the intrinsic invasiveness of embryos, deep interstitial implantation depends on active migration of preDeC and encapsulation of the conceptus (102–104). In 2D and 3D co-culture experiments, migratory preDeC first home in and then attach to the polar trophectoderm before “dragging” the conceptus into the stromal matrix (18, 104). This process is remarkable in several aspects. First, it is time sensitive as subsequent differentiation of preDeC into DeC leads to complete loss of directed migration and attachment to the conceptus. When extrapolated to the *in vivo* situation, these observations indicate that lack of senDeC, which accelerates the emergence of DeC, may lead to entrapment of the conceptus in a largely static matrix and implantation failure (18, 105). Second, high-quality human embryos stimulate migration of preDeC whereas low-quality embryos fail to do so. Conversely, migration of undifferentiated stromal cells is actively inhibited by high-quality embryos, but not low-quality embryos (104, 106, 107). Taken together, these observations suggest that the initial steps in the implantation process evolved in fundamental aspects across Eutherian (placental) mammalian species, likely reflecting maternal adaptations to different challenges imposed by rapidly evolving embryos (108). By relaxing the barrier function of the luminal epithelium, the implantation process arguably becomes primarily under control of preDeC cells, which first engage in embryo biosensing before actively encapsulating the conceptus or not.

### Embryo Biosensing and Selection

**Figure 2:**
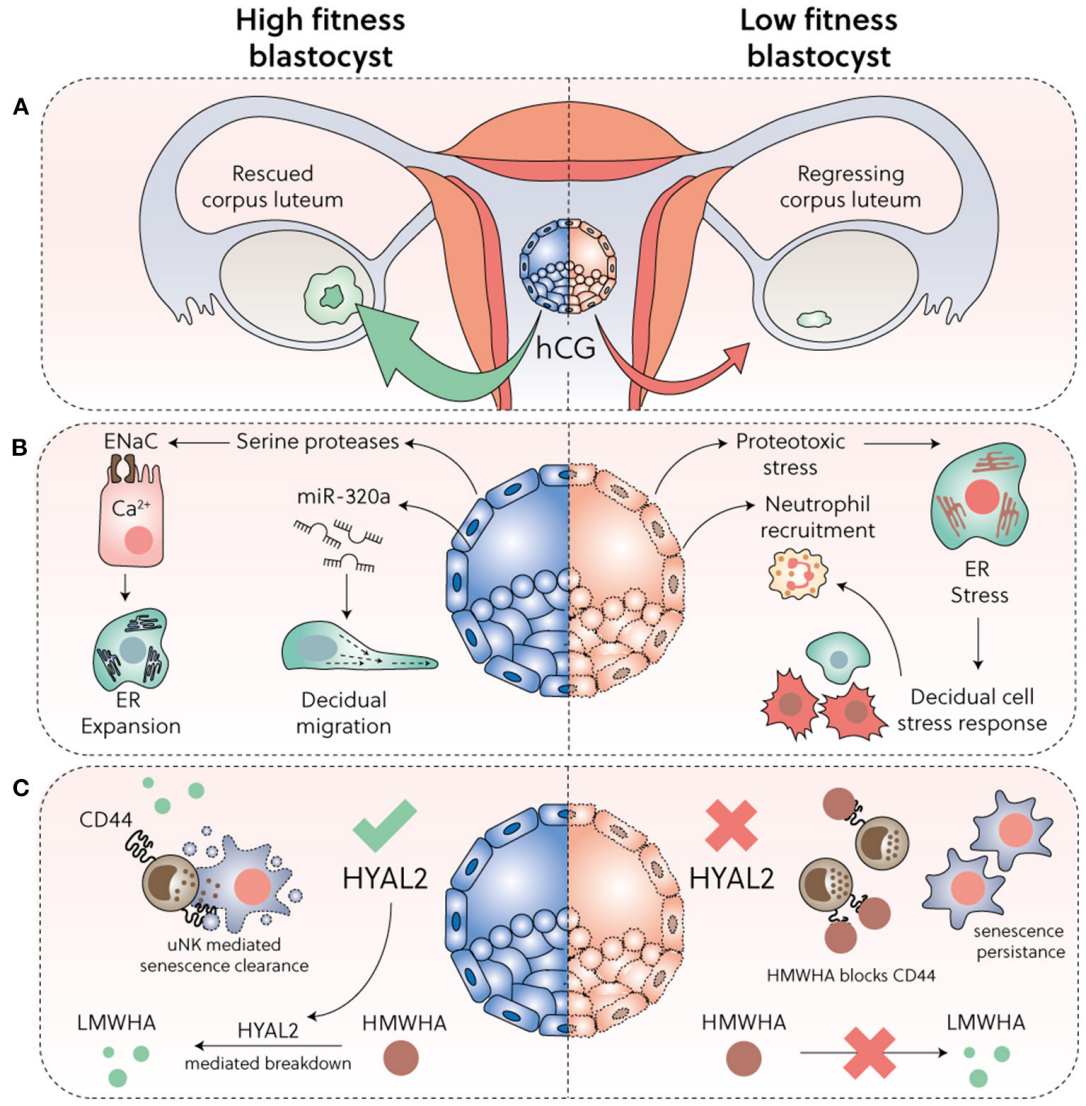
Embryo biosensing and selection. **(A)** Sufficient hCG secretion from implanting blastocysts is required to prevent corpus luteum involution. **(B)** High-fitness human blastocysts elicit a supportive decidual response, including secretion of evolutionary conserved serine proteases that activate epithelial Na^+^ channel (ENaC) expressed on luminal epithelial cells, triggering Ca^2+^ signalling, ER expansion and subsequent induction of implantation specific genes. Further, by secretion of microRNA miR-320a, competent blastocysts promote migration of pre-decidualizing cells. Conversely, low fitness embryos trigger a decidual ER stress response, repression of key implantation factors, and secretion of CXCL12 and CXCL8 leading to neutrophil recruitment and activation. **(C)** HYAL2 production in developmentally competent blastocysts cleaves HMWHA into LMWHA, which supports further development. Loss or reduction of HYAL2 in low-fitness embryos promotes HMWHA accumulation, which binds to CD44 expressed on uNK cells and disables selective killing of senDeC.

Figure 2 summarises the various mechanisms implicated in embryo biosensing by preDeC and DeC. While it is increasingly incontrovertible that spontaneous decidualization bequeaths the endometrium the ability to decode embryonic fitness signals (3, 109), how this process leads to menstruation-like disposal of developmentally compromised embryos is less obvious. Clearly, if the conceptus fails to secrete sufficient human chorionic gonadotropin (hCG), the corpus luteum involutes and falling progesterone levels trigger endometrial breakdown (Figure 2A). However, several prospective cohort studies in young, healthy women reported that 30% of pregnancies will fail after an initial rise in hCG levels (110–113). Most of these failures occur soon after implantation and therefore remain undetected. In a substantial number of early pregnancy losses, hCG concentrations only diverge from those in healthy pregnancies when the miscarriage is in progress (114), suggesting that an alternative mechanism of tissue breakdown is activated (Figures 2B,C). A recent study highlighted that embryonic fitness signals modulate the ability of uNK cells to target and clear senDeC. In this study, spent media from IVF embryos that subsequently failed to implant completely abrogated uNK cell-mediated killing of senDeC *in vitro* (37). Loss of embryonic hyaluronidase 2 (HYAL2) production was shown to be responsible for uNK cell inhibition (Figure 2C). HYAL2 cleaves high molecular weight hyaluronic acid (HMWHA) into low molecular weight hyaluronic acid (LMWHA) (115, 116). Hyaluronic acid, a ubiquitous ECM glycosaminoglycan, exerts distinct biological effects dependent on its molecular weight. In pre-implantation embryos, high HYAL2 activity and LMWHA production promotes development, whereas HMWHA does the opposite (116). Upon implantation, binding of HMWHA to CD44 expressed on uNK cells abrogates targeted killing of senDeC, whereas LMWHA has no inhibitory effect. Addition of recombinant HYAL2 to the spent medium of low-fitness human blastocysts was sufficient to restore uNK cell-mediated clearance of senDeC, at least *in vitro* (37). On the other hand, hCG promotes proliferation uNK cells (117), which presumably enhances immune surveillance of senDeC in conception cycles. Thus, the timing of embryo disposal after implantation is likely determined by the balance of opposing fitness signals as well as the ability of the endometrium to decode these signals, a function that resides with DeC and uNK cells.

## Endometrial Breakdown and Repair

In non-conception cycles, falling progesterone levels disable cooperation between DeC and uNK cells, which steers the decidual pathway to senDeC and transDeC involved in tissue breakdown and repair, respectively. Notably, senescent cells also co-opt innate immune cells, foremost neutrophils and macrophages, which upon activation and degranulation reinforces cellular senescence and ECM breakdown (2, 118). As outlined above, low-fitness embryos also disrupt uNK cell-DeC interactions, thereby engineering their own demise by triggering menstruation-like breakdown (Figures 2B,C). Thus, a switch in decidual state towards senDeC and transDeC may be the common pathway underpinning tissue breakdown and repair in menstruation and early pregnancy loss.

During menstruation, areas of shed endometrium are found alongside areas of unshed and repaired tissue (1, 67). This appearance of the endometrium during menstruation reinforces our conjecture that promulgation of cellular senescence primes the superficial endometrial layers for tissue breakdown, which upon recruitment of leucocytes becomes irrevocable. Arguably, a piecemeal approach to menstrual shedding abates the risk of infection and excessive haemorrhage, although rapid repair of the luminal epithelial remains critical (2). The mechanism driving re-epithelization of the endometrium during menstruation is, however, contentious. Based on scanning electron microscopy (SEM) studies, re-epithelization was initially attributed to proliferating or migratory epithelial cells arising from exposed gland stumps in the basal layer or from residual intact epithelium near the cornual and isthmic regions of the uterus (67, 119). Subsequent studies refuted this interpretation as there is no evidence of cellular proliferation during menstrual repair (120). Further, the discovery of isolated or small islands of immature epithelial cells with a smooth surface and a low cuboidal shape by SEM indicated that re-epithelization is primarily driven by differentiation of stromal cells via MET (1, 120). More recently, identification of ambiguous cells that express both epithelial and mesenchymal marker genes in luteal phase endometrium and decidualizing assembloids by single-cell transcriptomics provides additional credence to the assertion that MET (i.e., transDeC) play an important role in endometrial re-epithelization following menstrual desquamation (Figure 1B) (8, 18). Of course, both mechanisms are not mutually exclusive.

The mechanism that protects the regenerative basal layer from menstrual shedding is not entirely clear but presumably reflects the lack of hormone responses in this layer. Further, endometrial glands form a horizontal network in the basal layer, which may confer protection against menstrual destruction and aid regeneration (121). Intriguingly, transient but not prolonged exposure to SASP promotes tissue rejuvenation by reprogramming committed cells into stem-like cells (122). In fact, dedifferentiation of resident cells into stem cells is now recognised as the dominant mechanism for tissue regeneration in multiple organs (123). These observations raise the intriguing possibility that premenstrual senescence determines the regenerative potential of the basal layer following menstruation. In other words, the capacity of cells to cope with replication stress during oestrogen-dependent hyperproliferation may already be determined by the level of premenstrual senescence in the preceding cycle. Thus, while premenstrual senescence may be important for inter-cycle homeostasis, prolonged exposure to SASP, for example associated with clinical miscarriages, is predicted to impact adversely on stemness of the basal layer and to increase the likelihood of endometrial dyshomeostasis in subsequent cycles.

## Decidual Dyshomeostasis and Recurrent Miscarriage

### The Recurrence Risk of Miscarriage

Recurrent miscarriage is a devastating disorder and a sentinel risk factor for obstetrical disorders in future pregnancies (124). Approximately 15% of all clinically recognised pregnancies end in miscarriage, mostly before 12 weeks of gestation. The population prevalence of women with one, two or three or more previous miscarriages is 10.8, 1.9, and 0.7%, respectively. Apart from the physical trauma (pain, bleeding, and infection), each miscarriage compounds the risk of significant psychological morbidity (depression, post-traumatic stress disorder, and suicide) and obstetrical complications in a future ongoing pregnancy (preterm birth, foetal growth restriction, placental abruption, and stillbirth) (124).

Two independent risk factors, maternal age and the number of previous pregnancy losses, have disproportional effects on miscarriage rates (125, 126). The age-related risk is driven by meiotic errors in oocytes leading to foetal aneuploidy and increases sharply after the age of 34-years. Lack of geographic or ancestry-related variation indicates that the age-related risk is “hardwired” in human reproduction (127). Miscarriage rates also increase stepwise by ~10% with each additional loss (125, 126). This recurrence risk is age-independent and therefore not driven by chromosomal errors. To date, there is no epidemiological evidence that the recurrence risk of miscarriage has changed in recent decades nor does it differ substantially between populations (128–130). Thus, as is the case for age-related risk of miscarriage, the recurrence risk may also be grounded in a fundamental (patho-)physiological process, plausibly triggered by the miscarriage itself.

Clinically, recurrent miscarriage is defined by an arbitrary number of previous, consecutive or non-consecutive, pregnancy losses (124, 131). There is no consensus on precise criteria, although both the American Society for Reproductive Medicine (ASRM) and the European Society of Human Reproduction and Embryology (ESHRE) now define recurrent miscarriage as two previous pregnancy losses. The losses do not have to be consecutive, but the ESHRE definition includes preclinical (biochemical) losses whereas the ASRM definition does not (124). Importantly, there is no pathophysiological rationale for these definitions as the risk of miscarriage increases stepwise, even after a single loss, in women of all ages (125, 126, 128–130). Further, arbitrary definitions of recurrent miscarriage increase the risk of amalgamation of patients with wildly different prognoses under a single disease umbrella. For example, after two consecutive pregnancy losses, recurrent miscarriage patients aged 30 or 40 years will have ~80 and ~53% chance, respectively, of a successful next pregnancy (125). Hence, even over this age range, it is more likely than not that a subsequent pregnancy will be successful in these recurrent miscarriage patients, although women aged 40 will also have to content with reduced fertility. After 5 consecutive losses, however, the likelihood of a pregnancy resulting in live birth drops to ~55 and ~25%, respectively, in women aged 30 and 40 years (125). Thus, binary definitions of recurrent miscarriage are a major confounder in research and clinical trials as the likelihood of reproductive success and the effectiveness of therapeutic interventions differ markedly between study populations (132).

It is standard practise to attribute the recurrence risk of miscarriage to a host of subclinical disorders, ranging from subtle clotting, endocrine and immunological perturbations to vitamin deficiency and lifestyle factors. Apart from progesterone support, this disease paradigm has not resulted in effective interventions that prevent miscarriages, even after decades of research and numerous clinical trials (131–134). Further, two large cohort studies reported no difference in the likelihood of a successful pregnancy in women with “explained” vs. “unexplained” (“idiopathic”) recurrent miscarriage (135, 136). Despite the astonishing lack of evidence, clinical practise remains grounded in a historical, and arguably patriarchal, misconception that early pregnancy represents an exceptionally precarious physiological state, easily disrupted by “perturbations” that otherwise do not impact overtly on health and well-being outside pregnancy (137). This disease paradigm does not explain high cumulative live birth rates in miscarriage patients, nor is it easily reconciled with the incontrovertible fact that all our ancestors, over millennia, reproduced successfully despite harsher environments and poorer health conditions. Of course, overt disease, such as uncontrolled diabetes, can cause early pregnancy loss but cases are rare. Further, our criticism of current clinical practise does not challenge the notion that certain risk factors, such as obesity, impact adversely on the prognosis of miscarriage patients by aggravating the underlying pathophysiology (124, 131, 138).

### The Peri-implantation Endometrium in Recurrent Miscarriage

Surprisingly little attention has been paid to implantation biology in the context of recurrent miscarriage. The discovery that nidation is critically controlled by decidual subsets with opposing functions under the homeostatic control of extra-uterine innate immune cells and bone marrow derived decidual progenitors point towards a novel disease dimension. Recurrent miscarriage is associated with loss of clonogenicity in midluteal endometrium (76), reflecting lack of decidual progenitors (14). Based on computational modelling, decidual progenitors are predicted to give rise in early pregnancy to a distinct subset of cells present the superficial compact layer of the decidua (decidua compacta), that is, the site of initial trophoblast invasion (14). Importantly, the level of stem cell depletion in midluteal endometrium correlates inversely with the number of previous pregnancy losses and, hence, the recurrence risk of miscarriage (76). Several studies have focused on the abundance of uNK cells in luteal phase endometrium of recurrent miscarriage patients (139), invariably motivated by the questionable assumption that high levels signal a pending immune attack on the semi-allogenic conceptus (140). However, the abundance of uNK cells varies naturally throughout the luteal phase and between cycles (13), which is entirely in keeping with the homeostatic role of these innate immune cells. Lack of standardised protocols and failure to normalise uNK cell levels for cycle day further accounts for inconsistent findings in the literature (139). Nevertheless, there is evidence that lower uNK cell levels and activity in the endometrium, as well as peripheral blood, associates with higher miscarriage rates (8, 141–143). Together, the data suggest that key homeostatic mechanisms that regulate the transformation of the cycling endometrium into the decidua of pregnancy are relaxed in recurrent miscarriage patients. Loss of stringency is predicted to increase the likelihood of embryo implantation in an endometrium that is destined for breakdown in early pregnancy and, by extension, the recurrence risk of miscarriage. For example, obesity adversely impact on decidual progenitor cells in peri-implantation endometrium, exemplified by a significant inverse correlation between increased body mass index and the level of clonogenic endometrial cells (144). Obesity is further associated with uNK cell depletion and dysfunction in pregnancy (145), which may explain why it is a major risk factor for higher-order miscarriages (146).

**Figure 3:**
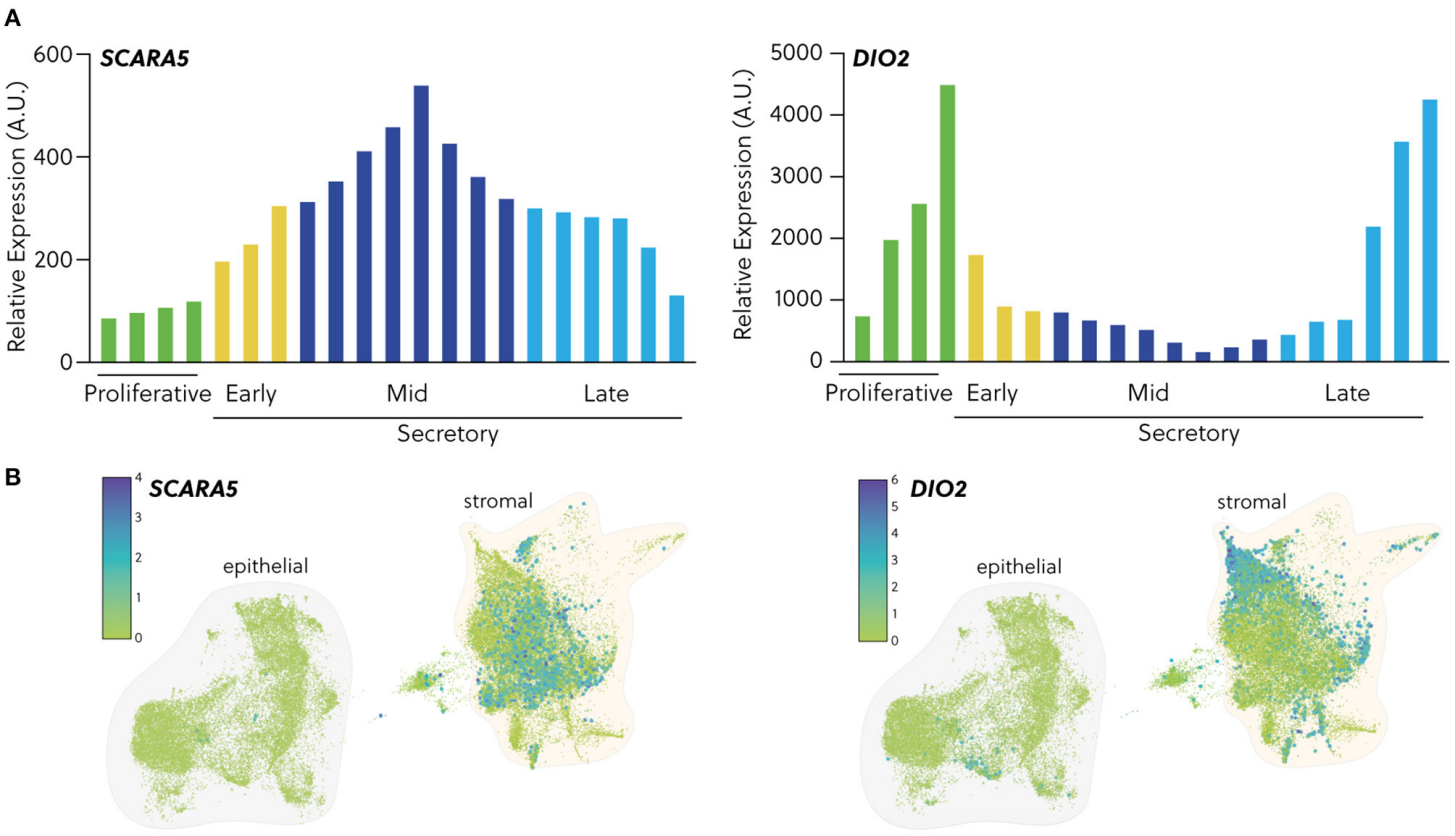
Expression of cell fate biomarkers. **(A)** *In silico* analysis of GDS2052 microarray data showing transcript expression of the DeC biomarker *SCARA5* and the senDeC biomarker *DIO2* in proliferative, early-, mid- and late-secretory endometrium expressed in arbitrary units. **(B)** Temporal expression of *SCARA5* and *DIO2* transcripts in human endometrium from single cell profiling of endometrial biopsies obtained across the cycle (*n* = 15). Data were extracted from Garcia-Alonso et al. (148) and available on reproductivecellatlas.org.

Perturbations in the peri-implantation endometrium can potentially be exploited to identify clinically useful biomarkers for screening of women at increased risk of miscarriage before pregnancy. Such biomarkers may also be useful in assessing pre-pregnancy interventions aimed at optimising the uterine implantation environment. Accurate assessment of endometrial clonogenicity is possible but cumbersome as it relies on colony-forming unit assays that take 10 days to complete (76, 144, 147). Measurements of uNK cell levels are also fraught because of intrinsic intra- and inter-cycle variations (13). An alternative approach is to measure the relative abundance of different decidual subsets in midluteal biopsies. An early sign of stromal cells earmarked for cellular senescence in pregnancy is lack or loss of progesterone-responsiveness, defined here as expression of genes firmly repressed by progesterone. For example, progesterone strongly represses *DIO2*, a stromal cell-specific gene in the endometrium that encodes iodothyronine deiodinase 2, the enzyme that catalyses the conversion of prohormone thyroxine (T4) to the bioactive thyroid hormone (T3) (8). As shown in Figure 3A, *DIO2* expression is high during the proliferative phase and the late-secretory phase, i.e., when progesterone levels are low and stromal cells are highly metabolically active. *SCARA5*, encoding the ferritin receptor, is a stromal cell-specific, progesterone-responsive gene (8). Not unexpectedly, the temporal expression profile of *SCARA*5 across the menstrual cycle is the inverse of that of *DIO2*. As cells cannot be simultaneously progesterone-responsive and -resistant, *SCARA5* and *DIO2 a priori* mark distinct stromal subpopulations in peri-implantation endometrium (Figure 3B). Interestingly, recurrent pregnancy loss is associated with increased frequency of cycles with low *SCARA5* and high *DIO2* expression in midluteal biopsies (8), indicating that lack of DeC at implantation predisposes for senescence-mediated breakdown of the placental-decidual interface in pregnancy. In agreement with this conjecture, a recent single-cell transcriptomic study reported a striking senescence-associated gene signature in stromal and decidual cells at the maternal-foetal interface upon the diagnosis of missed miscarriage (149), indicating that decidual senescence precedes the physical disintegration of pregnancy.

## Summary and Therapeutic Perspective

Single-cell “omics” approaches are rapidly transforming our understanding of the cellular dynamics underpinning key endometrial functions, including embryo implantation, menstrual shedding and repair, and the spectacular transformation of a short-lived uterine mucosa into a robust matrix that accommodates the placenta throughout pregnancy. Underpinning all these functions is spontaneous decidualization, an iterative process that follows positional E2-dependent endometrial hyperproliferation and leads to emergence of subsets of cells with specialised functions that control endometrial fate decisions at implantation. Balancing decidual subsets and states from one cycle to the next is under control of non-uterine cells. On the one hand, uNK cells engender selective elimination of senDeC, *de facto* rejuvenating the endometrium following embryo implantation (37). On the other, recruitment and engraftment of bone marrow-derived decidual progenitor cells may impart tissue plasticity to accommodate a rapidly growing conceptus and invading trophoblast (14).

Recent insights in the cellular dynamics during the peri-implantation window are poised to lead to bespoke therapeutic interventions for intractable reproductive disorders, such as recurrent miscarriage. Based on our current understanding, two therapeutic “windows” in the menstrual cycle are predicted to have maximal impact on an ensuing pregnancy. First, interventions could focus on enhancing the stringency of homeostatic control in peri-implantation endometrium. For example, a recent study demonstrated that bone marrow transplants from wild-type mice to mice carrying a heterozygous deletion of *Hoxa11*, a pivotal decidual transcription factor, not only restore the decidual response but also prevent pregnancy loss in these animals (16). Further, sitagliptin, a dipeptidyl-peptidase IV (DPP4) inhibitor used in the management of diabetes, was found in a randomised, double-blind placebo-controlled feasibility trial to increase the abundance of decidual progenitor cells by almost 70% when given over 3 consecutive menstrual cycles to recurrent miscarriage patients (147). Notably, increased engraftment of bone marrow-derived progenitors coincided with a marked reduction in *DIO2* expression in peri-implantation endometrium, suggesting amelioration of the pro-senescence endometrial state. There is yet no information on whether the endometrial effects of sitagliptin are transient or durable. Recruitment of decidual progenitors and uNK cells likely continues at the maternal-foetal interface in early gestation, at least until the second trimester of pregnancy when transformation of maternal spiral arteries into large fibrinoid vessels by endovascular trophoblast is complete (11, 150). Hence, it seems sensible to maintain treatment after embryo implantation, but sitagliptin is not licenced for use in pregnancy, at least in the UK, because of insufficient safety data. An alternative approach to balance decidual subpopulations is to target E2-dependent hyperproliferation. Therapeutic interventions confined to the proliferative phase would go a long way in abating justifiable fears of embryo/foetal toxicity of novel drugs. This strategy is not only appealing but increasingly realistic, especially in view of current explosion in the development of drugs that target stressed and senescent cells for the treatment of age-related disorders (63, 151). These drugs can be broadly categorised into two categories: pharmacological agents termed “senolytics,” which eliminate senescent cells, and “senomorphics,” which prevent the detrimental cell-extrinsic effects of senescent cells and include SASP inhibitors (63, 151). Further, organoid and assembloid technologies now enable rapid screening of the effectiveness of drugs in restoring or enhancing the implantation environment (18, 152–154). Altogether, a new age of non-hormonal “endometrial therapeutics” appears just around the corner.

## Author Contributions

JB conceptualised the article. JB, JM, and C-SK drafted the article. JM prepared the figures. All authors approved the final version.

## Funding

The Tommy's Charity funds the Tommy's National Centre for Miscarriage Research. JJB is a holder of a Wellcome Trust Investigator Award (Grant/Award Number: 212233/Z/18/Z), University Hospital Coventry and Warwickshire (UHCW) NHS Trust provided further financial support.

## Conflict of Interest

The authors declare that the research was conducted in the absence of any commercial or financial relationships that could be construed as a potential conflict of interest.

## Publisher's Note

All claims expressed in this article are solely those of the authors and do not necessarily represent those of their affiliated organizations, or those of the publisher, the editors and the reviewers. Any product that may be evaluated in this article, or claim that may be made by its manufacturer, is not guaranteed or endorsed by the publisher.
